# Supplementary material for: Ultra-narrow room-temperature emission from single CsPbBr3 perovskite quantum dots
Source: Nat Commun. 2022 May 11;13:2587. doi: 10.1038/s41467-022-30016-0 (PMC9095639; doi:10.1038/s41467-022-30016-0)
Supplement: Supplementary file 1 — Supplementary Information [file 41467_2022_30016_MOESM1_ESM.pdf]

SUPPORTING INFORMATION

Ultra-Narrow Room-Temperature Emission from Single $CsPbBr_3$ Perovskite Quantum Dots

Gabriele Rainò[1,2,*], Nuri Yazdani[3], Simon C. Boehme[1,2,4], Manuel Kober-Czerny[1,2], Chenglian Zhu[1,2], Franziska Krieg[1,2], Marta D. Rossell[5], Rolf Erni[5], Vanessa Wood[3], Ivan Infante[4,6], Maksym V. Kovalenko[1,2*]

[1]Institute of Inorganic Chemistry, Department of Chemistry and Applied Biosciences, ETH Zurich, 8093 Zurich, Switzerland

[2]Laboratory for Thin Films and Photovoltaics, Empa − Swiss Federal Laboratories for Materials Science and Technology, CH-8600 Dübendorf, Switzerland

[3]Department of Information Technology and Electrical Engineering, ETH Zurich, Zurich, 8092 Switzerland

[4]Department of Theoretical Chemistry, Faculty of Science, Vrije Universiteit Amsterdam, 1081 HV Amsterdam, The Netherlands

[5]Electron Microscopy Center, Empa – Swiss Federal Laboratories for Materials Science and Technology, CH-8600 Dübendorf, Switzerland

[6]Department of Nanochemistry, Istituto Italiano di Tecnologia, Via Morego 30, 16163, Genova, Italy

*Correspondence to: rainog@ethz.ch, mvkovalenko@ethz.ch

Supplementary Note 1

Computational methods

QD construction: The cubic QDs are cut directly from bulk cubic-$CsPbBr_3$. As noted below, we fully geometry relax all atoms of the QDs, and find that the atomic structure of the QDs post-geometry-relaxation is independent of whether the initial crystal structure is cubic or orthorhombic[1]. The eight Cs atoms on the corner of the cubic QDs are then removed. The overall charge balance of the QDs is given by[2],

$$N_{Cs} + 2N_{Pb} - N_{Br} + N_{ch} = 0,$$

where N_x refers to the number of Cs, Pb, or Br atoms, or surplus/deficiency of additional charges (*ch*). To maintain overall charge balance, we add additional electrons to the QDs. Such charges could, in reality, be donated through bonding with surface ligands, or counter ions in solution, neither of which we treat explicitly here. This approach, as opposed to the removal of a fraction of surface Cs atoms, maintains the overall cubic symmetry of the QDs, greatly facilitating our analysis, and allowing a direct comparison of QD sizes, without any additional stochastic effects.

DFT/AIMD calculations: Our general approach to the density-functional theory (DFT) and *ab-initio* molecular dynamics (AIMD) calculations was to specifically favor the computation of realistically sized QDs at a lower level of theory over the computation of unrealistically small QDs at a higher level of theory.[3,4] Specifically, we have employed the single-particle instead of a many-body description of the bandgap transition,[5] use the Perdew-Burke-Ernzerhof (PBE) functional, apply the harmonic approximation, and omit spin-orbit coupling. Such a choice regarding the considered QD size and level of theory, proved, *a posteriori*, well justified by a very good match with the experimental observations (see main text).

Geometry optimization, electronic structure calculations, and ab initio molecular dynamics (AIMD) were carried out utilizing the CP2K program suite along with the quickstep module[6]. A dual basis of localized Gaussians and plane-waves were used for the calculations[7], along with a 300 Ry plane-wave cutoff. Goedecker–Teter–Hutter pseudopotentials[8] for core electrons, and the Perdew–Burke–Ernzerhof (PBE) exchange correlation functional are used for all calculations, with an enforced convergence to 10^{-8} in Self-Consistent Field calculations. Cubic unit cells with dimensions {$(5.0\ \text{nm})^3$, $(6.0\ \text{nm})^3$, $(7.0\ \text{nm})^3$, $(7.5\ \text{nm})^3$, $(7.5\ \text{nm})^3$} are defined for the {1.8 nm, 2.4 nm, 3.0 nm, 3.6 nm, and 3.6 nm core-shell} QD respectively.

Prior to AIMD, full geometry optimizations are performed on all QDs, relaxing all atoms of each system. This is accomplished using the Quickstep module utilizing a Broyden–Fletcher–Goldfarb–Shannon (BFGS) optimizer, with a maximum force of 24 meVÅ^{-1} used as convergence criteria.

AIMD was performed within the canonical ensemble using a CSVR thermostat[9]. AIMD time steps of 10 fs are used. For the 3.6 nm NCs and smaller, 3 ps of the AIMD are run with the time constant of the thermostat (τ_{th}) set to τ_{th} = 15 fs for thermalization, calibration, and equilibration of the total energy and temperature. This is followed by 10 ps run of the AIMD with τ_{th} = 1 ps. Post-processing of the atom trajectories are performed, removing the six macroscopic degrees of freedom (3x translation, 3x rotation)

using the Iterative Closest Point algorithm[10]. For the 4.2 nm QD, AIMD is initially run for 2 ps with a τ_{th} = 15 fs at 300 K. τ_{th} is then set to 100 fs, and after 1 additional ps of AIMD @ 300K, the temperature of the thermostat is set to 10 K. After the temperature of the system equilibrated at 10 K (~5 ps), τ_{th} was set to 1 ps for data collection.

Due to the size of the QDs calculated here, we are not able to include spin-orbit coupling, which is required in order to accurately capture the fine structure of the lowest energy optical transition of CsPb-halide perovskite QDs[11]. Calculations and experiments have demonstrated fine splitting of the lowest energy transition of up to ~5 meV. Our neglect of spin-orbit interaction can thus lead to errors in the computed linewidth on this order, a small correction.

Phonon density of states: We compute the phonon density of states of the QD $g(\omega)$, and partial density of states for each atom, $g_i(\omega)$,

$$g_i(\omega) = m_i\omega^2|\mathcal{F}\{r_i(t)\}|^2 \frac{\hbar\omega}{k_BT}\left(1 + \left(e^{\hbar\omega/k_BT} - 1\right)^{-1}\right), \quad g(\omega) = \sum_i g_i(\omega) \qquad (1)$$

where ω is the phonon frequency, m_i is the mass of atom i, $r_i(t)$ is the atomic trajectory, $\hbar$ Planck's constant over 2π, k_B the Boltzmann constant, and T the temperature, and $\mathcal{F}\{x\}$ corresponds to the Fourier transform of x.

Electron-phonon coupling calculations within the harmonic approximation: Within the harmonic approximation, the dimensionless Huang-Rhys factor $S_{ij,\omega}$ represents the coupling of a phonon/vibration with frequency ω to a transition from the state ψ_i to the state ψ_j[12], and is related to the shift in the normalized equilibrium atomic coordinates ($Q_{i\omega}$ and $Q_{j\omega}$ for the states ψ_i and ψ_j), along the direction of the vibration, $S_{ij,\omega} = \frac{1}{2}(Q_{j\omega} - Q_{i\omega})^2$ [13]. For a particular transition, temperature dependent rates of both radiative and non-radiative transitions can be determined given all $S_{ij,\omega}$ for each phonon with frequency ω which couples to the transition[14]. A spectral representation of the complete set of $S_{ij,\omega}$, $S_{ij}(\omega_k)$, can be computed directly from the adiabatic energy surfaces extracted from an AIMD simulation of a finite system[15].

Each vibrational degree of freedom has an average energy of k_BT in a semiclassical AIMD simulation. Each vibrational mode will then have a time-dependent normal coordinate given by:

$$Q_\omega(t) = \sqrt{\frac{2k_BT}{\hbar\omega}}e^{i\omega t+\phi} + Q_{0\omega} \qquad (2)$$

where $Q_{0\omega}$ is the equilibrium normal coordinate in the system's ground state. The energy of state ψ_i, again within the harmonic approximation, is given by,

$$E_i(Q) = E_i + \sum_\omega \frac{1}{2}\hbar\omega(Q_\omega - Q_{i\omega})^2 \quad (3)$$

where the sum is over all vibrational modes and $Q_{i\omega}$ is the equilibrium normal coordinate in state ψ_i. From eq. 1 and 2, the time-dependent energy of the transition $\psi_i \rightarrow \psi_j$ is:

$$E_j(t) - E_i(t) = (E_j - E_i) + \sum_\omega \left[\frac{1}{2}\hbar\omega\left(Q_\omega(t) - Q_{j\omega}\right)^2 - \frac{1}{2}\hbar\omega(Q_\omega(t) - Q_{i\omega})^2\right]$$

$$= (E_j - E_i) + \sum_\omega\left(\sqrt{S_{0i,\omega}S_{ij,\omega}} + \sqrt{S_{0j,\omega}S_{ij,\omega}}\right)\hbar\omega + \sum_\omega \sqrt{4S_{ij,\omega}\hbar\omega k_B T}e^{i\omega t+\phi} \quad (4)$$

The first and second terms correspond to the energy difference and Stokes shift between states E_i and E_j. The third term is the only time dependent one, and we can extract $S_{ij}(\omega_k)$ through a Fourier transform, $\mathcal{F}\{\dots\}$, of the time dependence of the transition's energy from the AIMD trajectory[15],

$$S_{ij}(\omega_k) = \left|\mathcal{F}\{E_j(t) - E_i(t)\}\right|^2/4\hbar\omega k_{\mathrm{B}}T \quad (5)$$

The number of time steps of the AIMD run determines the spectral resolution of $S_{ij}(\omega_k)$. This is a spectral representation of the $S_{ij,\omega}$ as the extracted $S_{ij}(\omega_k)$ at some particular ω_k is the sum of $S_{ij,\omega}$ within the frequency range $(\omega_k - 1/2\Delta\omega, \omega_k + 1/2\Delta\omega)$ where $\Delta\omega = \omega_k - \omega_{k-1}$.

In practice, extracting $S_{ij}(\omega_k)$ with this approach is best done with very low temperature AIMD simulations for two reasons (1) it is derived within the harmonic approximation, at high temperatures anharmonic motion may be present which would then contribute to the computed $S_{ij}(\omega_k)$ (eq. 2) at elevated temperatures, the time dependent energies of both states may likely have avoided crossings of the adiabatic levels. In order to use eq. 5 one must be able to track the adiabatic energy of both states uniquely. For this reason, in both Main Text and Supporting Information, $S_{ij}(\omega_k)$ was always extracted from AIMD simulations at 10 K. Since we furthermore only consider interband transitions between valence band maximum (VBM, HOMO, state ψ_i) and conduction band minimum (CBM, LUMO, state ψ_j), in the following and in the main text we simplify the notation to:

$$S_\omega \equiv S_{VBM,CBM}(\omega_k)$$

In Supplementary Figure 4, we plot the computed phonon density of states for each of the QDs investigated in the main text, in addition to the computed electron-phonon coupling strengths to the

lowest-energy transition. The coupling strengths, integrated over the energy ranges presented in Fig. 3 of the main text, *i.e.*

$$S_{\langle\omega\rangle} = \frac{1}{\langle\omega\rangle}\sum_{\omega_{min}}^{\omega_{max}} S_{\omega_i} \cdot \omega_i\,, \quad \langle\omega\rangle = \frac{\omega_{max}+\omega_{min}}{2} \tag{6}$$

are given in Supplementary Table 1.

For the $CsPbBr_3/CsCaBr_3$ core/shell QDs, the high energy Ca-Br vibrations (~25 meV) show negligible coupling, as expected for the type-I heterostructure. We stress that the $CsPbBr_3/CsCaBr_3$ is a robust type-I core-shell structure, even in the presence of the dynamics. For example, if we consider the electron density in the CBM of the QD, we find only ~1.7% is within the orbitals of the Ca atoms and ~67% within Pb orbitals (note: there are 152 Ca atoms in the shell and 64 Pb atoms in the core). The electron is thus quite strongly confined to the QD core, as it is also highlighted by the molecular orbital plot of Fig. 2e in the main text. Once the dynamics are turned on, this density does indeed fluctuate, but only very little (see Supplementary Figure 5). We therefore concluded that even in the presence of the dynamics, the electron and hole both remain strongly confined to the QD core.

Temperature dependence of the linewidth and electron-phonon coupling calculations at room temperature: To investigate further the temperature dependence of the linewidth, we performed additional temperature-dependent AIMD simulations for the ~2.4 nm QD presented in the main text, covering a range T = 10 K to T = 300 K. We note that both the harmonic approximation discussed above (sideband broadening via phonon replicas, with the temperature dependence given in eq. 2 of the main text) and anharmonic interactions (*i.e.* decoherence due to phonon-phonon scattering, and anharmonic lattice distortions) can all contribute to the linewidth at room temperature. As shown in the following, the total broadening resulting from all interactions can again be directly extracted from the adiabatic energy surfaces generated by the AIMD.

Using the optical response formalism[16], we compute the time- and temperature-dependent dephasing function $D(t,T) = e^{-g(t,T)}$, where $g(t,T)$ is the lineshape function

$$g(t,T) = \frac{1}{\hbar^2}\int_0^t d\tau_1 \int_0^{\tau_1} d\tau_2 C(\tau_2,T) \tag{7}$$

which is obtained from $C(\tau_2,T) = \langle \Delta E_g(\tau_2,T)\Delta E_g(0,T)\rangle$, the autocorrelation function of the bandgap fluctuation $\Delta E_g(\tau_2,T) = E_g(\tau_2,T) - \langle E_g(\tau_2,T)\rangle$. Square brackets $\langle \dots \rangle$ denote time-averaging. The gap fluctuation and autocorrelation function are shown in Supplementary Figure 6a and 6b, respectively. As shown in Supplementary Figure 6c, the dephasing function for all temperatures is well described by a

Gaussian function with standard deviation σ_t, representing the pure-dephasing time t_2^*. The latter allows us to directly extract the FWHM of the emission line according to

$$\Gamma(T) = 2\sqrt{2\ln(2)}\frac{\hbar}{t_2^*} \quad (8)$$

In Supplementary Figure 6d, we plot the extracted linewidth as a function of temperature using equation (2) of the main text, $\Gamma(T) = 2\sqrt{2\ln(2)}\sqrt{2\Lambda k_B T}, \Lambda = \sum_\omega S_\omega \hbar\omega$ (dashed line), and the linewidth extracted directly from the AIMD using eq. 5 above (points). While at low temperatures the extracted linewidths are in excellent agreement with those estimated from S_ω within the harmonic approximation, divergences start to occur at temperatures > 50 K. This suggests there are additional contributions to the room temperature linewidth, such as enhanced anharmonic ion mobility of atoms about the QD surface. Before delving into this further in the next sections, we note one important point: at room temperature, the phonon-sideband broadening accounts for 70% of the overall linewidth. Hence, phonon sideband broadening resulting from coupling to low energy phonons on the QD surface does indeed dominate the overall linewidth over the broad temperature range up to 300 K.

Frozen-shell AIMD simulations: To further validate surface contributions to emission line broadening in $CsPbBr_3$ QDs, we distinguish core and surface atoms by describing a 2.9 nm $CsPbBr_3$ QD as if comprised of a $CsPbBr_3$/$CsPbBr_3$ core/shell QD. We now further impose a mechanical distinction by allowing the core atoms to freely move, while freezing the shell atoms. This "mechanical" distinction between core and surface (both comprised of $CsPbBr_3$) is analogous to the common "electronic" core/shell structures where core and shell are defined via differing materials, and thus different electronic structures. One notable difference is that in our mechanical core/shell structure the electronic wavefunctions can still delocalize over the shell region.

As illustrated in the inset of Supplementary Figure 7a, we construct QDs with shell thicknesses of 0.5 (red), 1 (ochre), 1.5 (green), and 2 perovskite unit cells (blue), respectively, corresponding to shell thicknesses of about 0.3, 0.6, 0.9, and 1.2 nm, respectively. In other words: the frozen shell comprises the outermost CsBr layer, CsBr + $PbBr_2$ layers, CsBr + $PbBr_2$ + CsBr layers, and CsBr + $PbBr_2$ + CsBr + $PbBr_2$ layers, respectively.

Supplementary Figure 7a depicts the results of our AIMD simulation at 300 K. Upon freezing progressively thicker shell layers, we find a clear reduction of the HOMO and LUMO fluctuations. Supplementary Figure 7b shows the correspondingly reduced band gap (LUMO-HOMO) fluctuations,

with associated histograms akin to experimental PL line-broadening spectra. Gaussian fits to the data yield a FWHM of 230 meV for a completely free QD. Upon progressively freezing the outer (shell) layer, the FWHM decreases significantly, down to a mere 50 meV for a QD with a frozen shell of 2 unit-cell thickness. Such a large (five-fold) reduction in FWHM demonstrates the effectiveness of immobilizing surface atoms. A type-I core/shell heterostructure (see main text Fig. 4) constitutes a promising route for realizing narrow band-emission, as it reduces wavefunction overlap with the large-amplitude surface vibrations (see main text Fig. 2f).

Frozen-core AIMD simulations: Here, we present additional calculations on the origin of the increased coupling to low- and medium-energy vibrational modes for small QDs (see main text Fig. 2d), and specifically their relation to the QD surface. To this end, we study several frozen core/free shell $CsPbBr_3$/$CsPbBr_3$ QD, *i.e.* the inverse structure as compared to Supplementary Figure 7. Supplementary Figure 8a shows the exciton-phonon coupling spectrum for several of such QDs (see inset). By gradually freezing a thicker core region, we move from the entirely free QD (grey) to a more "surface"-like structure, eventually arriving at a QD with a free shell of only 0.5 unit cells (red), where only the outermost CsBr atoms are contributing to electron-phonon coupling. To enable a fair comparison of all partially frozen structures, we normalize the 'effective' Huang-Rhys factor S_ω to the number of free atoms. We refer to it as 'effective' S_ω as it is obtained from AIMD simulations at 300 K, hence potentially including some anharmonic contribution (unlike the results presented in the main text, computed in the fully harmonic case at 10 K). Freezing the core (and hence increasing the surface contribution) yields a progressively stronger contribution by low- and medium energy modes, indicated by the red and green shaded areas centered at 4 and 8 meV, respectively. The observed trend (summarized in Supplementary Figure 8b) suggests that the coupling to low- and medium-energy modes is enhanced for the surface. This is in excellent agreement with the same conclusion derived from the size-dependent emission linewidth reported in Fig. 2d of the main text. Summarizing, by freezing the QD core and separately studying the surface contribution to exciton-phonon coupling, we establish that the QD surface contributes significantly to emission broadening via an enhanced coupling to low- and medium-energy phonon modes.

Enhanced mobility of surface atoms: To study the atomistic origin for the enhanced emission linewidth at elevated temperatures (Supplementary Figure 6d), in Supplementary Figure 9 we report the root mean square fluctuation ρ_i^{RMSF} of atomic positions $\boldsymbol{r}_i(t)$ in a 2.9 nm $CsPbBr_3$ at 300 K during an AIMD simulation (trajectory length = 4.25 ps, time step = 5 fs), given by

$$\rho_i^{RMSF} = \sqrt{\langle(\boldsymbol{r}_i - \langle\boldsymbol{r}_i\rangle)^2\rangle} \quad (9)$$

where i is the atom index and square brackets denotes time averages. Supplementary Figure 9a shows such fluctuations for each of the 775 atoms in the 2.9 nm QD. The color coding distinguishes the different elements and follows the sketch in the inset. The blue highlighted regions indicate each atom's distance to the surface: with respect to the CsBr surface layer (dark blue), the subsequent $PbBr_2$, CsBr, and $PbBr_2$ layers are located - 0.5, -1.0, and - 1.5 unit cells (u.c.) below the surface, respectively. In general, Cs and Br are more mobile than Pb, in line with the reported A-site polar fluctuations[17] and rotational disorder[18], respectively. Furthermore, these large fluctuations monotonously increase towards the surface: Supplementary Figure 9b shows that surface Br is 40 % more mobile than Br atoms located at 1.5 unit cells below the surface. Such an enhanced ion mobility at the surface is also found in PbS QDs, where it broadens the PL linewidth and accelerates nonradiative decay[19]. In PbS QDs, the soft surface extends about 1 nm into the QD. In our $CsPbBr_3$, the ion mobility still has not reached a plateau at 1.5 unit cells (*i.e.* 0.9 nm) below the surface. Overall, these MD simulations clearly suggest that reducing the large mobility of surface atoms in $CsPbBr_3$ QDs, up to at least 1 nm below surface, represents a promising route to decrease the emission linewidth.

Supplementary Note 2

Scanning transmission electron microscopy analysis: Processing of the averaged ADF-STEM images was performed in MATLAB, using custom-developed scripts[20,21]. Background intensity variations were removed by performing a morphological opening using a structuring element[22]. A disk structuring element with a sufficiently large radius (*i.e.*, a few interatomic distances) was used and the obtained result was smoothed with a 2D Gaussian filter having the same radius as the structuring element. Thus, we were able to extract the background of the image, which contained the low spatial frequencies information due to the carbon support. The background was subtracted from the raw data to obtain the background-corrected signal, which was then denoised using a custom-developed nonlinear filtering algorithm based on the method proposed by H. Du[23]. The obtained results provided the denoised and background-corrected datasets. Next, deconvolution of the images was carried out in order to eliminate the effect of the finite lateral size of the electron probe[24]. We made use of a conventional blind deconvolution algorithm (MATLAB's "*deconvblind*" function) with an initial estimate of the point-spread function calculated from the microscope's parameters (*i.e.*, acceleration voltage and probe convergence semi-angle). The Cs and Pb/Br atomic columns positions in the ADF-STEM images were

fitted by means of a center of mass peak-finding algorithm, and subsequently refined by solving a least-squares minimization problem using the Levenberg-Marquardt algorithm. This iterative refinement was carried out using a custom-developed script that makes use of 7-parameter two-dimensional Gaussians[20,25]. Finally, the mean intensity and standard deviation values of the Cs and Pb/Br atomic columns were calculated and used to determine the Cs : Pb/Br intensity ratio.

Supplementary Figures 11 and 12 depict the analysis carried out for four core-only and four core/shell $CsPbBr_3$ QDs, respectively. The figures show, from top to bottom, the averaged ADF-STEM images of the QDs, the corresponding intensity maps of the Cs and the Pb/Br atomic columns and the Cs : Pb/Br intensity ratio. The mean Cs : Pb/Br intensity ratio of all original $CsPbBr_3$ QDs is 0.33 ± 0.02, while for the surface-modified QDs is 0.37 ± 0.01. Additionally, the ADF-STEM images distinctly show the presence of an amorphous shell of ~1 nm thickness for the surface-modified QDs, while no amorphous shell is detected in the original QDs. All these results suggest that the surface-modified QDs are depleted of Pb/Br as compared to the original $CsPbBr_3$ QDs. In order to rule out that the detected differences are a consequence of thickness induced dynamic scattering effects due to geometry differences between the original and the modified QDs, we performed ADF-STEM simulations at discrete crystal thicknesses. Additionally, a lattice spacing estimation for the core-only and core/shell $CsPbBr_3$ QDs was performed by means of peak-pair analysis[26]. Supplementary Figure 14 depicts a quantitative analysis of the lattice parameters for a core-only and a core/shell QD along two orthogonal (horizontal and vertical) directions. In both cases a mean lattice spacing of 0.6 nm is found, suggesting that the core/shell QDs do not have a built-in strain upon surface modifications.

Scanning transmission electron microscopy simulations: Simulations of the high-resolution ADF-STEM signals were performed using a custom-developed FORTRAN 77 program[27]. The simulations were calculated setting the parameters to the values used in the experiments, *i.e.* accelerating voltage: 300 kV; convergence semi-angle: 18 mrad; electron energy spread: 0.8 eV; effective geometrical source size: 0.25 Å; ADF angular range: 35-190 mrad; Gaussian blurring with sigma 0.3 Å[28]. A thickness series of $CsPbBr_3$ imaged along the [001] projection was calculated for 2 to 30 unit-cell crystals (1.17 to 17.61 nm) in 2 unit-cell increments using a slice thickness of half a unit cell. The simulation results are depicted in Supplementary Figure 13 and show a clear dependence of the atomic column intensity ratio on thickness. Thus, for crystal thicknesses of 15 and 30 unit cells, Cs : Pb/Br intensity ratios of ~ 0.370 and ~ 0.338 are obtained, respectively. These results provide evidence that the Cs : Pb/Br intensity ratio

differences observed between similarly sized original (0.33 ± 0.02) and surface-modified (0.37 ± 0.01) $CsPbBr_3$ QDs are necessarily caused by a depletion of Pb/Br in the latter.

size	S_{2meV}	S_{7meV}	S_{17meV}	Γ_{PL} (300K)
**1.8 nm**	29.9	8.4	1.2	200 meV
**2.4 nm**	19.7	6.6	0.5	164 meV
**3.0 nm**	11.0	4.4	0.8	138 meV
**3.6 nm**	7.0	1.9	1.7	126 meV
**4.2 nm**	7.5	1.0	1.8	122 meV
**Core/Shell**	5.3	1.3	0.5	89.9 meV

Supplementary Table 1. The computed electron-phonon coupling strength $S_{\langle\omega\rangle}$ to the lowest energy transition is given for each QD, along with the computed room temperature thermal broadening Γ_{PL}.

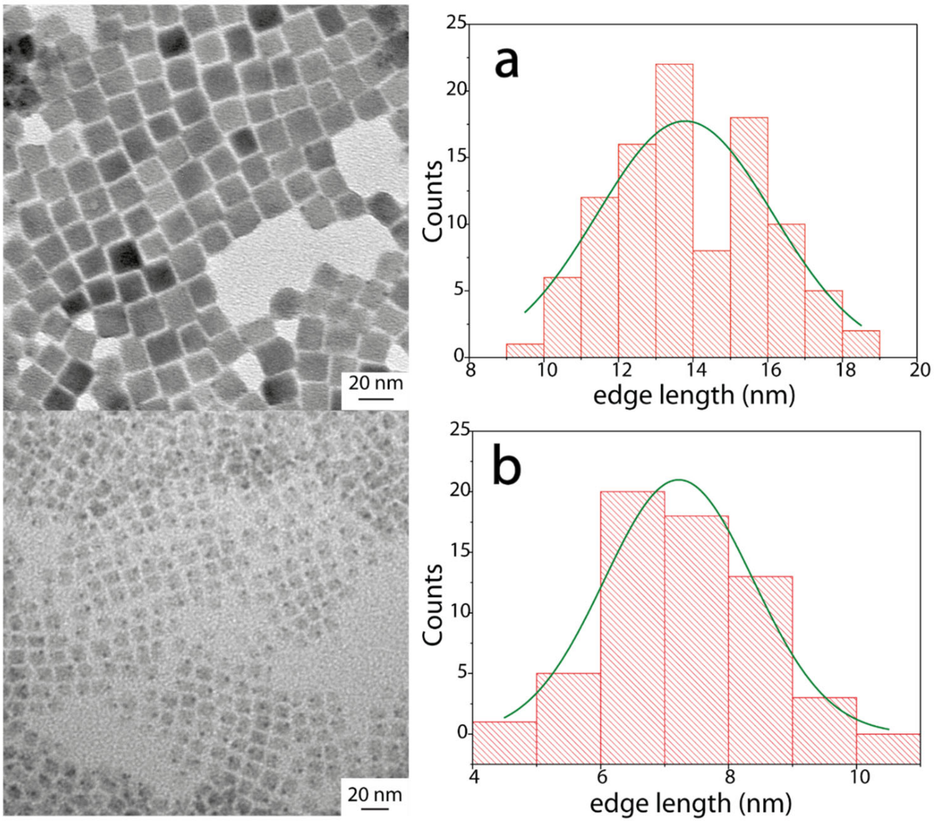

Supplementary Figure 1 | Statistical analysis of QD size distribution. Low magnification TEM images and the correspondent QD size distribution analysis reveals QD mean edge length of 14 nm in (**a**) and 7 nm in (**b**), respectively. In both cases, the standard deviation of QD size distribution is set around 15% (2 nm in **a**; 1 nm in **b**).

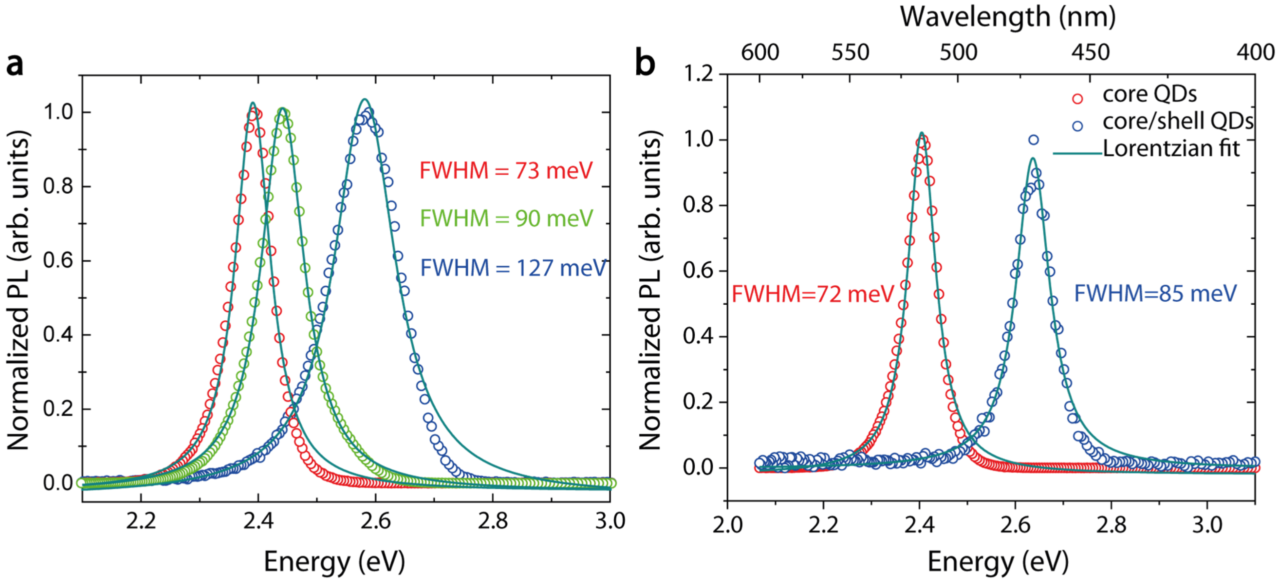

Supplementary Figure 2 | Ensemble PL spectra. a, Ensemble PL spectra for QDs with edge length 14 nm (red circle), 7 nm (green circle), 4.5 nm (blue circle). An increase in PL FWHM has been observed by reducing the QD size, from 73 meV up to 127 meV, in line with single QD spectroscopy results. **b,** ensemble PL spectrum before (red dot) and after (blue dots) dilution (x 12000). Solid lines are Lorentzian best fits of the experimental data. For core/shell QDs, the emission line broadening is much narrower than QDs emitting at the same energy (equivalent quantum confinement regime), in line with single QD spectroscopy results.

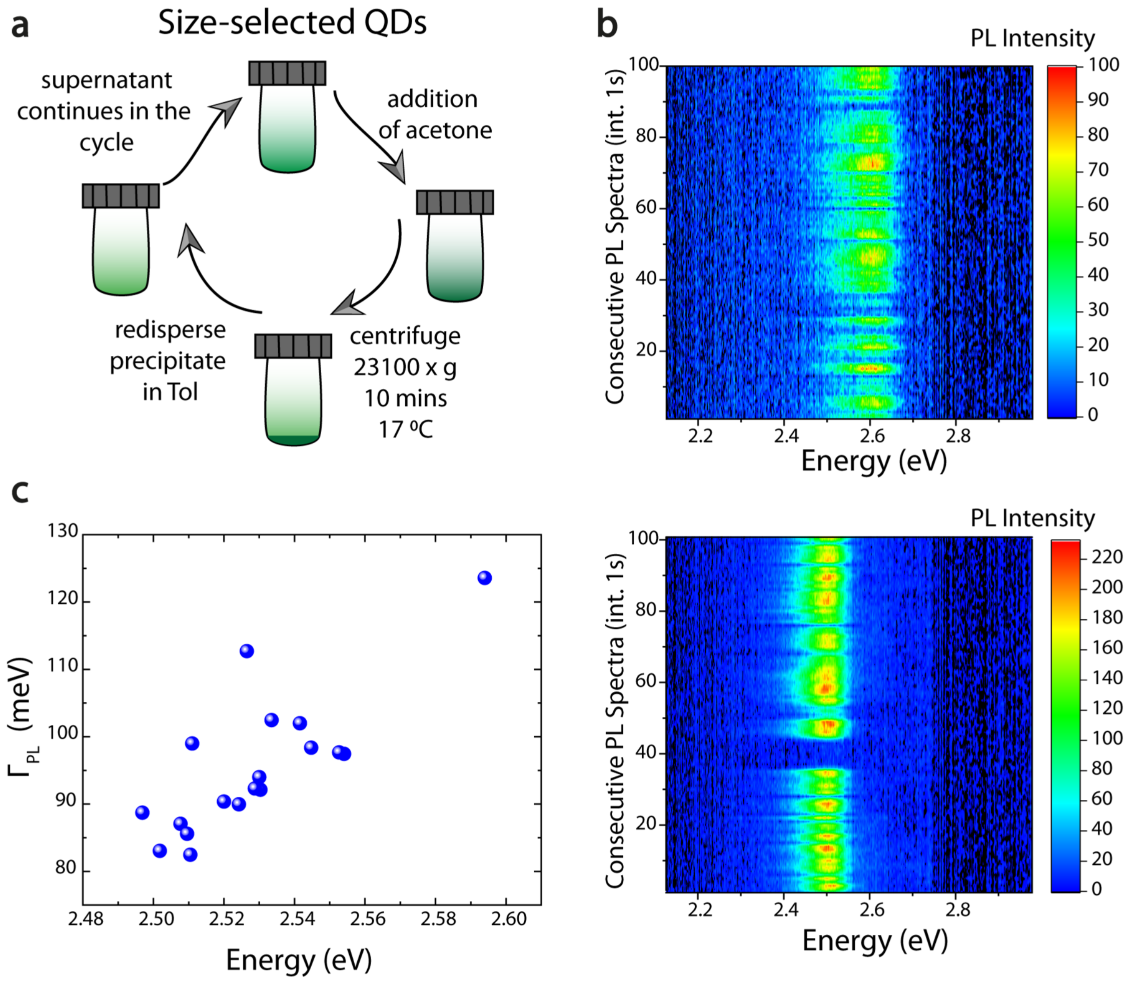

Supplementary Figure 3 | Size-dependent emission line-broadening in ultra-small QDs. a, Schematic of the size-selective precipitation method used to isolate colloids of ultra-small QDs (edge length 4-5 nm). Additional information can be found in ref.[29] **b,** The two-dimensional colored plots of several consecutive PL spectra obtained for two QDs emitting at 2.6 eV and 2.5 eV, respectively. Stable PL emission could only be achieved by encapsulating the QDs between two glass coverslips in inert (argon) atmosphere. **c**, Statistics of emission line-broadening for different QDs reveals a linear increase of emission broadening for higher emission energies, e.g. for smaller QDs.

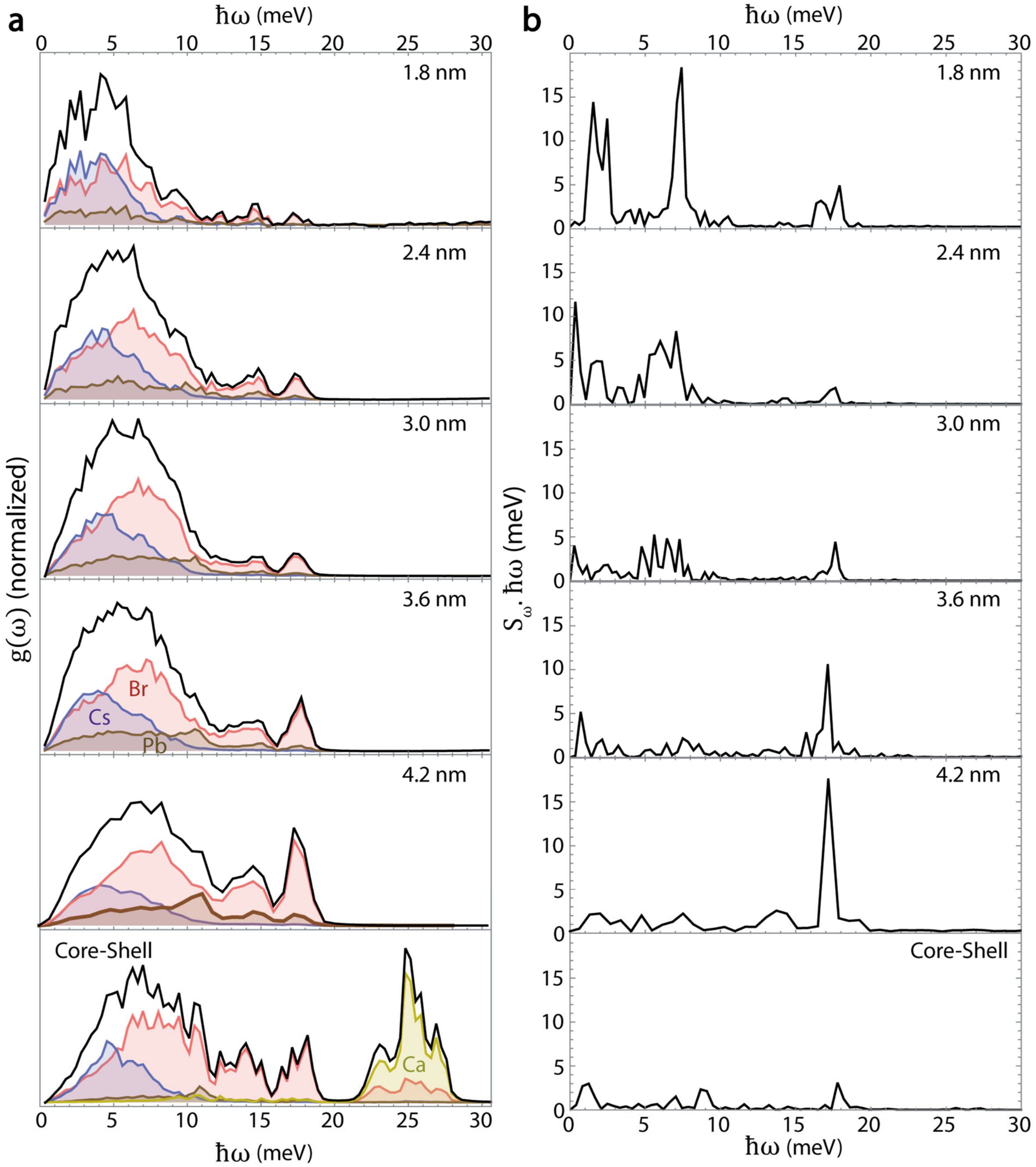

Supplementary Figure 4 | Calculated phonon density of states. a, Computed phonon density of states (black line), and partial density of states for each atomic species (colored filled lines) for all studied QDs. In **b**, the computed electron-phonon coupling strength to the lowest energy transition is plotted.

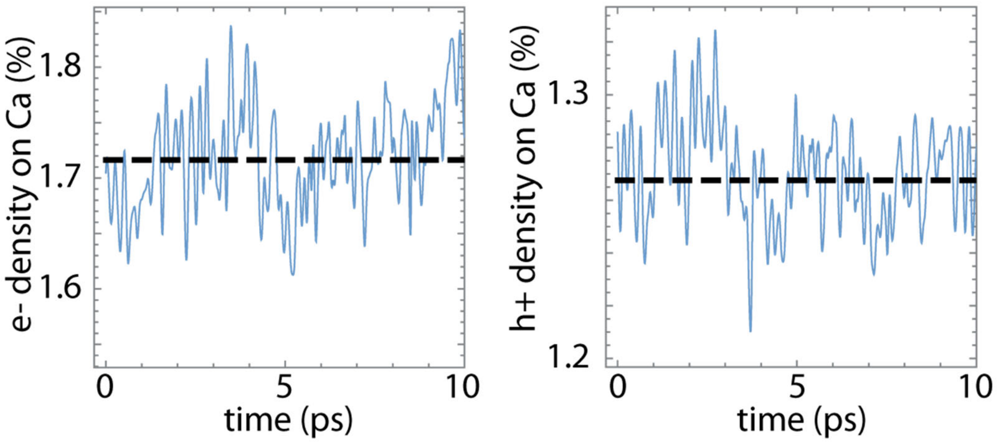

Supplementary Figure 5 | Electron/hole confinement by $CsCaBr_3$ shell. Electron density in the CBM on Ca orbitals, and hole density in the VBM on Ca orbitals during AIMD of the $CsPbBr_3/CsCaBr_3$ QD (blue line), and in the geometry relaxed QD (dashed line).

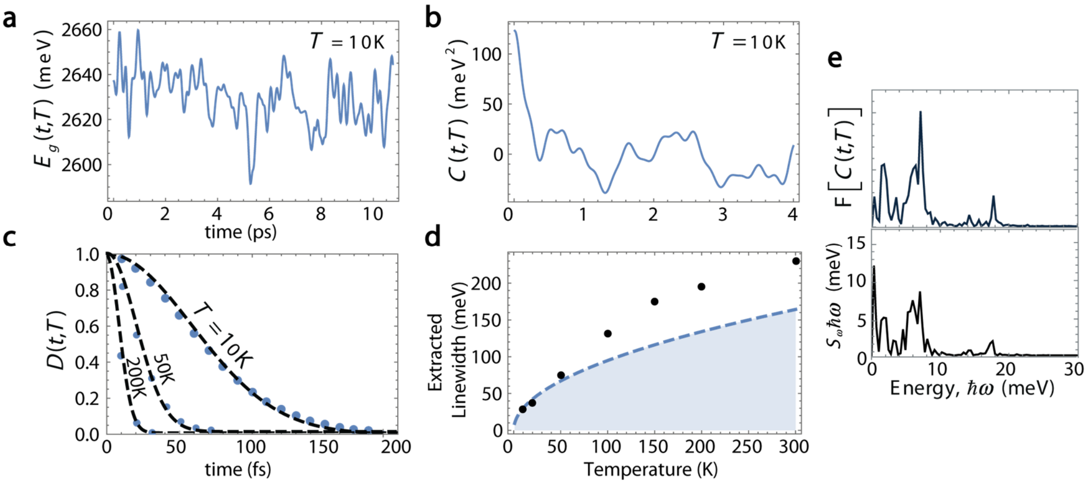

Supplementary Figure 6 | Temperature dependent linewidth. Temperature dependence of emission broadening in the 2.4 nm $CsPbBr_3$ QDs (as in the main text) employing the optical response formalism. **a-c**, show calculations while (**d)** displays the full temperature dependence from 10 K to 300 K. **a**, time-dependent bandgap fluctuation calculated via AIMD. **b**, autocorrelation function of the bandgap fluctuation. **c**, dephasing in the time domain (blue points), along with a Gaussian fit (black dashed line). **d**, FWHM of the emission line broadening (black points) resulting from the dephasing in (**c**), the blue dashed line represents the contribution to the linewidth from phonon emission/absorption computed with the S_ω presented in the main text. **e**, comparison of the spectral density obtained from the optical response formalism, $\mathcal{F}\{C(t,T)\}$, *i.e.* the Fourier transformation of the autocorrelation function depicted in **b**, with the spectrum of Huang-Rhys factors S_ω times $\hbar\omega$, as discussed in the main text. The similarity of both spectra visualize the equivalence of both analysis methods.

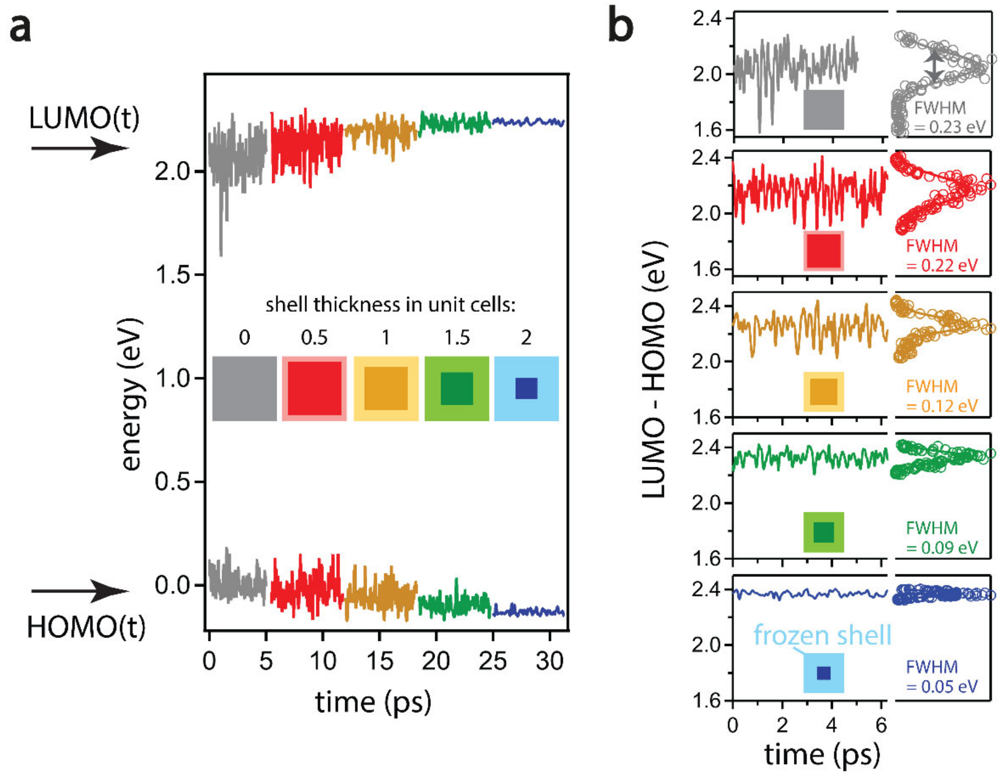

Supplementary Figure 7 | Emission linewidth in a 2.9 nm $CsPbBr_3$/$CsPbBr_3$ core/shell QD obtained from a 300 K AIMD simulation with a frozen shell. a, Starting from the entirely free QD (depicted in grey), the magnitude of the time-dependent fluctuations of the HOMO and LUMO decreases upon increasing the thickness of the frozen shell, from a thickness of 0.5 (red), 1 (ochre), 1.5 (green), up to 2 unit cells (blue). For clarity, traces are shifted in horizontal direction. **b**, Fluctuation of the HOMO-LUMO gap (left) and corresponding histogram (right) for the “free core/frozen shell” QDs introduced in (a). The decreases of the FWHM of the histogram suggests a reduced emission linewidth upon immobilizing surface atoms.

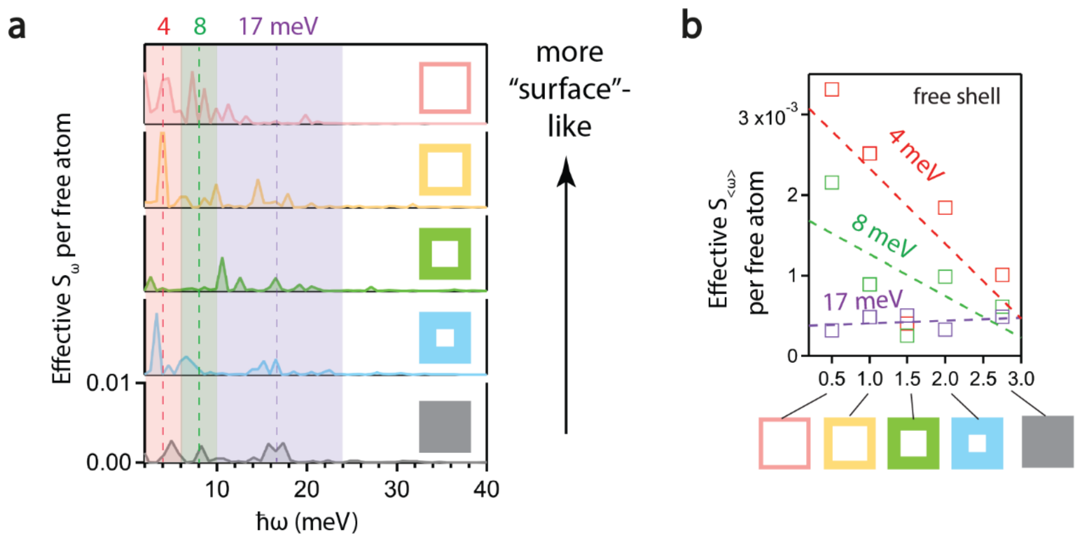

Supplementary Figure 8 | Effective Huang-Rhys factors S_ω obtained from a 300 K AIMD simulation with a frozen core. **a**, Effective S_ω per free atom for several 2.9 nm $CsPbBr_3/CsPbBr_3$ core/shell QDs with a frozen core (see inset for a schematic of the varying thickness of the free shells). The red, green, and purple shaded areas indicate low-, medium-, and high-energy modes around 4, 8, and 17 meV, respectively, as similarly introduced in the size-dependence analysis in Fig. 2c of the main text. **b**, Dependence of the effective $S_{<\omega>}$ per free atom on the thickness of the free shell, for the low-, medium-, and high-energy modes, depicted by red, green, and purple open markers, respectively. Linear fits serve as guide to the eye.

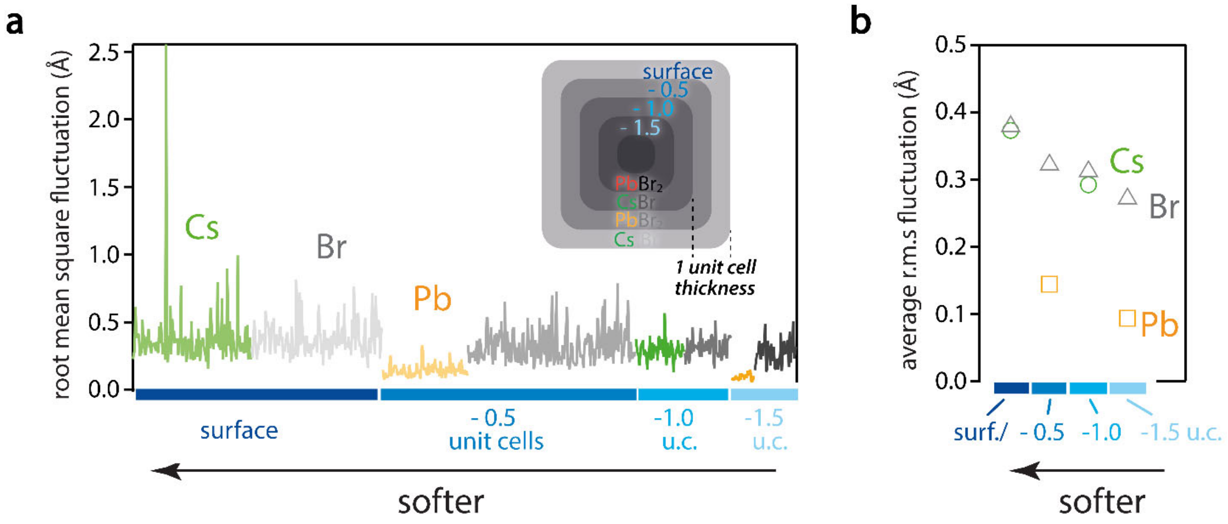

Supplementary Figure 9 | Very mobile surface atoms at the softer surface. Root mean square fluctuation of the nuclear positions of a 2.9 nm $CsPbBr_3$ QD, per atom (**a**), and averaged per atomic layer and element (**b**). The horizontal axis runs over atom indices i; blue-highlighted groups indicate the respective position in the QD (see inset), expressed in perovskite unit cells (u.c.) below the QD surface.

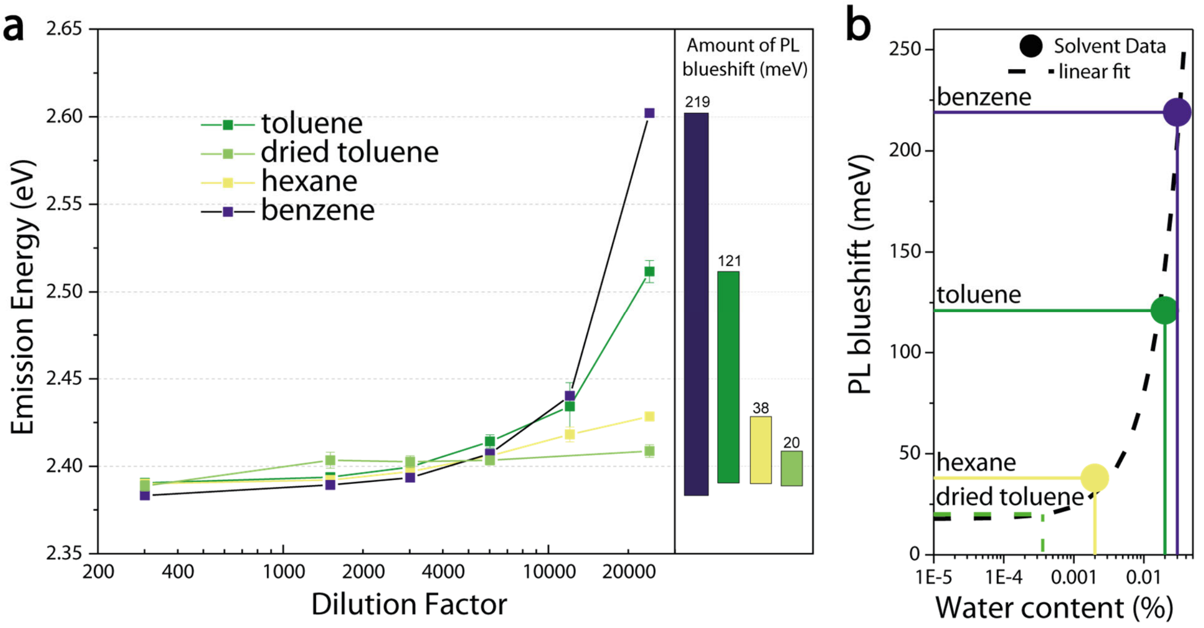

Supplementary Figure 10 | Dilution induced surface modifications: the role of the solvent. a, Dilution studies show the effect of different solvents on the dilution induced PL blueshift. On the right, the PL blueshift is measured as difference between the first (dilution factor 300) and the last emission peak energy (dilution factor 24 000). **b**, The PL blueshift (in meV) is plotted against the approximate water content (in %) of the three solvents, hexane, toluene and benzene (solid lines) as obtained from the manufacturers data sheets. Predicted water content (green dashed-lines) for dried toluene is in agreement with reported literature values[30].

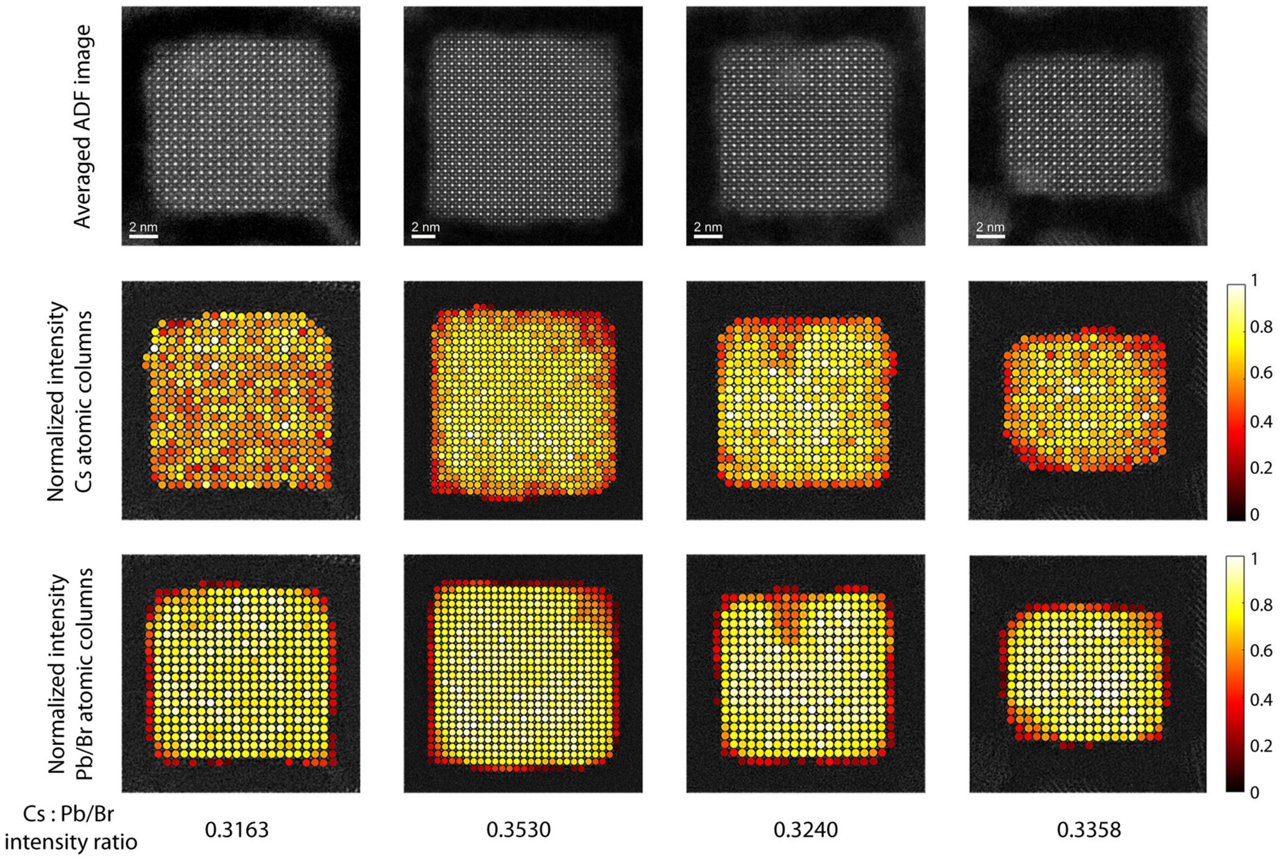

Supplementary Figure 11 | Structural analysis of core-only $CsPbBr_3$ QDs. ADF-STEM images of four original $CsPbBr_3$ QDs obtained through aligning and averaging images series consisting of 10 frames (1024x1024, 1 µs dwell time), and fit of the intensities of the Cs and Pb/Br atomic columns plotted at the fitted coordinates. For the sake of clarity and comparison purposes, the fitted intensities of each QD are normalized to the maximum atomic column intensity. The Cs : Pb/Br intensity ratio of each $CsPbBr_3$ QD is given below the corresponding QD, and the mean Cs : Pb/Br intensity ratio of all four QDs is 0.33 ± 0.02 (errors denote the standard deviation).

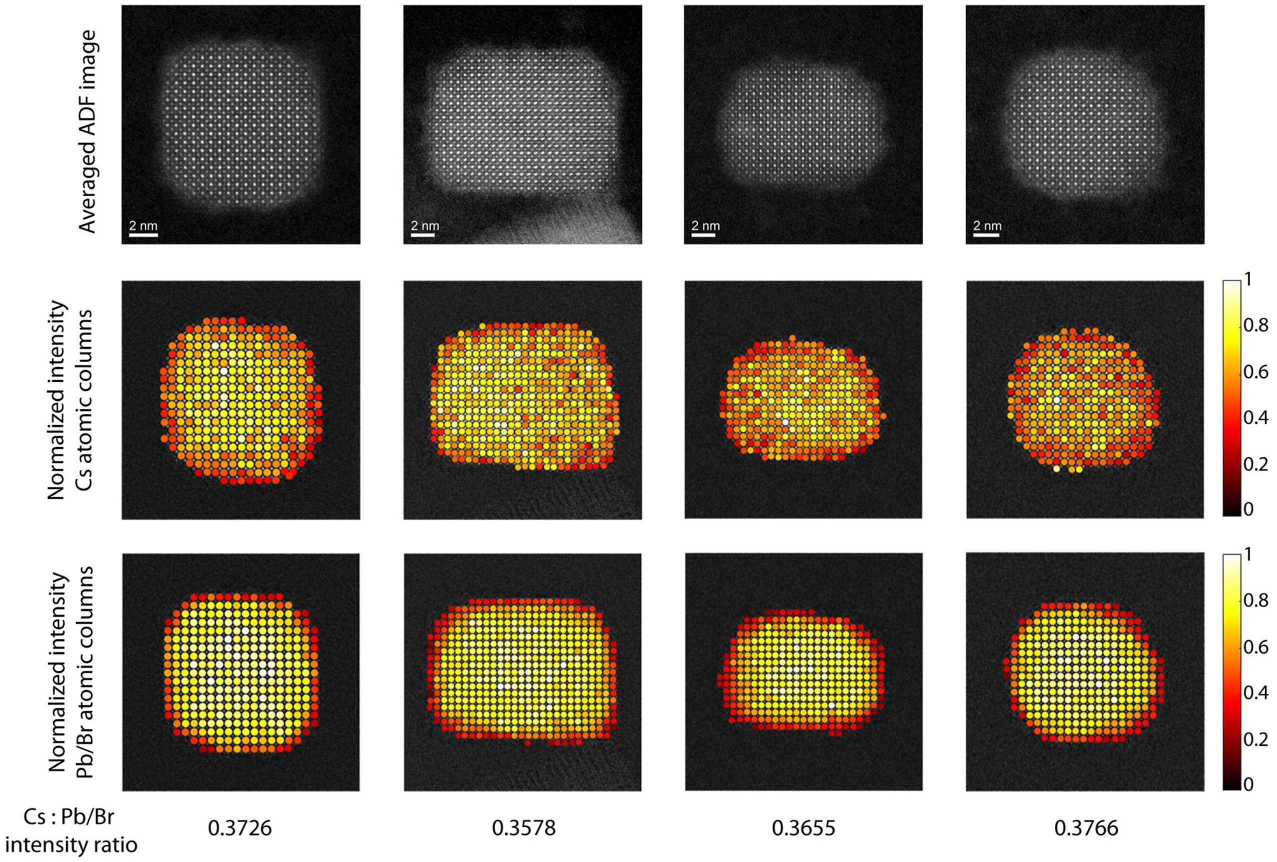

Supplementary Figure 12 | Structural analysis of surface-modified, core/shell QDs. ADF-STEM images of four core/shell $CsPbBr_3$ QDs obtained through aligning and averaging images series consisting of 10 frames (1024x1024, 1µs dwell time), and fit of the intensities of the Cs and Pb/Br atomic columns plotted at the fitted coordinates. For the sake of clarity and comparison purposes, the fitted intensities of each QD are normalized to the maximum atomic column intensity. The Cs : Pb/Br intensity ratio of each $CsPbBr_3$ QD is given below the corresponding QD, and the mean Cs : Pb/Br intensity ratio of all four QDs is 0.37 ± 0.01 (errors denote the standard deviation).

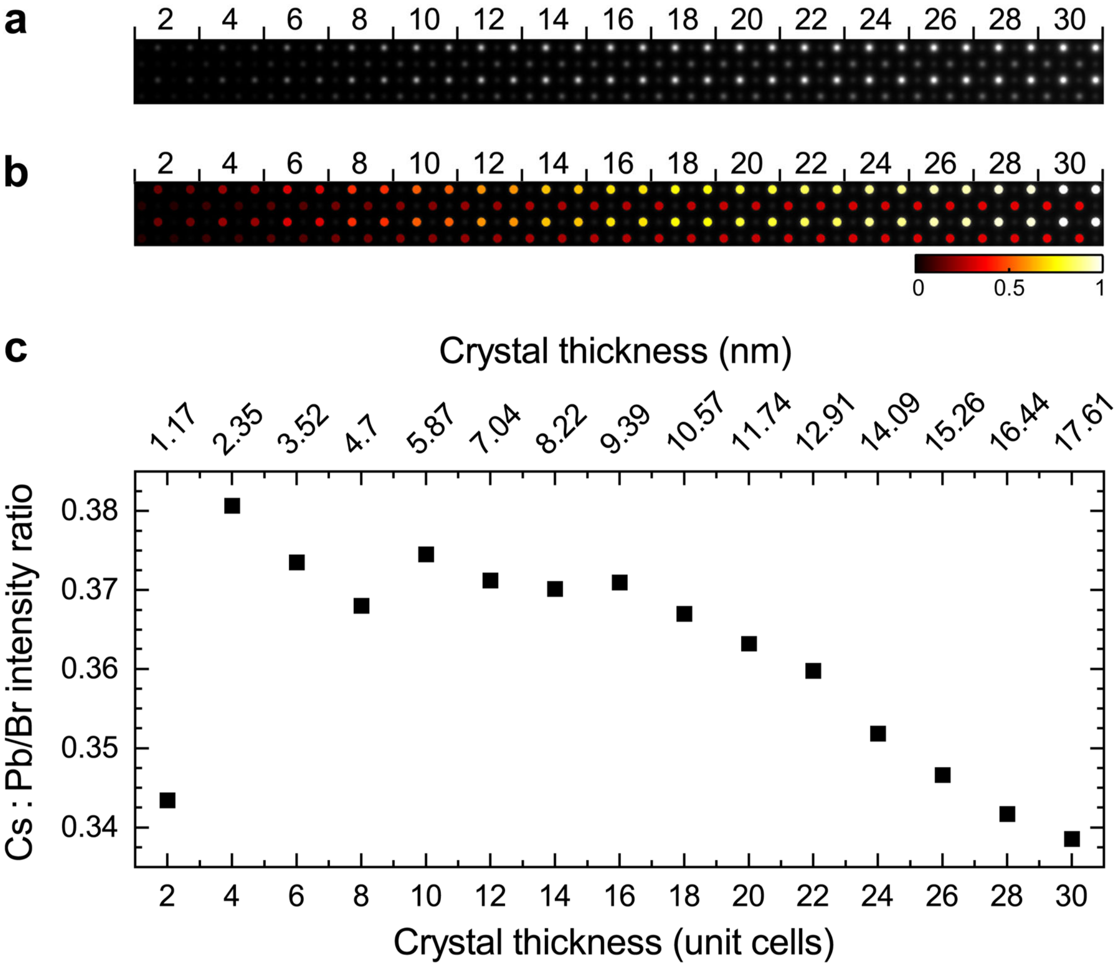

Supplementary Figure 13 | Calculated ADF-STEM images of $CsPbBr_3$. a, Simulated ADF-STEM images of $CsPbBr_3$ crystals with thicknesses increasing, from left to right, from 2 to 30 unit cells (1.17 to 17.61 nm), in 2 unit-cell increments. **b,** Extracted fit of the calculated intensities for the Cs atomic columns (second and fourth row) and Pb/Br atomic columns (first and third row) plotted at the fitted coordinates overlaid on the simulated ADF-STEM images. For clarity, the fitted intensities are normalized to the maximum atomic column intensity. **c,** Dependence of the atomic column intensity ratios on thickness obtained from the simulated ADF-STEM images. The comparison between experimental and simulated intensity ratio is qualitative, due to the fact that there is a known contrast difference between simulated and experimental electron microscopy data. In case of STEM simulations, this so called Stobbs-factor is usually attributed to be caused by the effective source size that is used as a fitting parameter to eventually match experimental and simulated image contrast. Because the experimental data were averaged and deconvolved, no further attempts were made to refine the effective source size in the simulations.

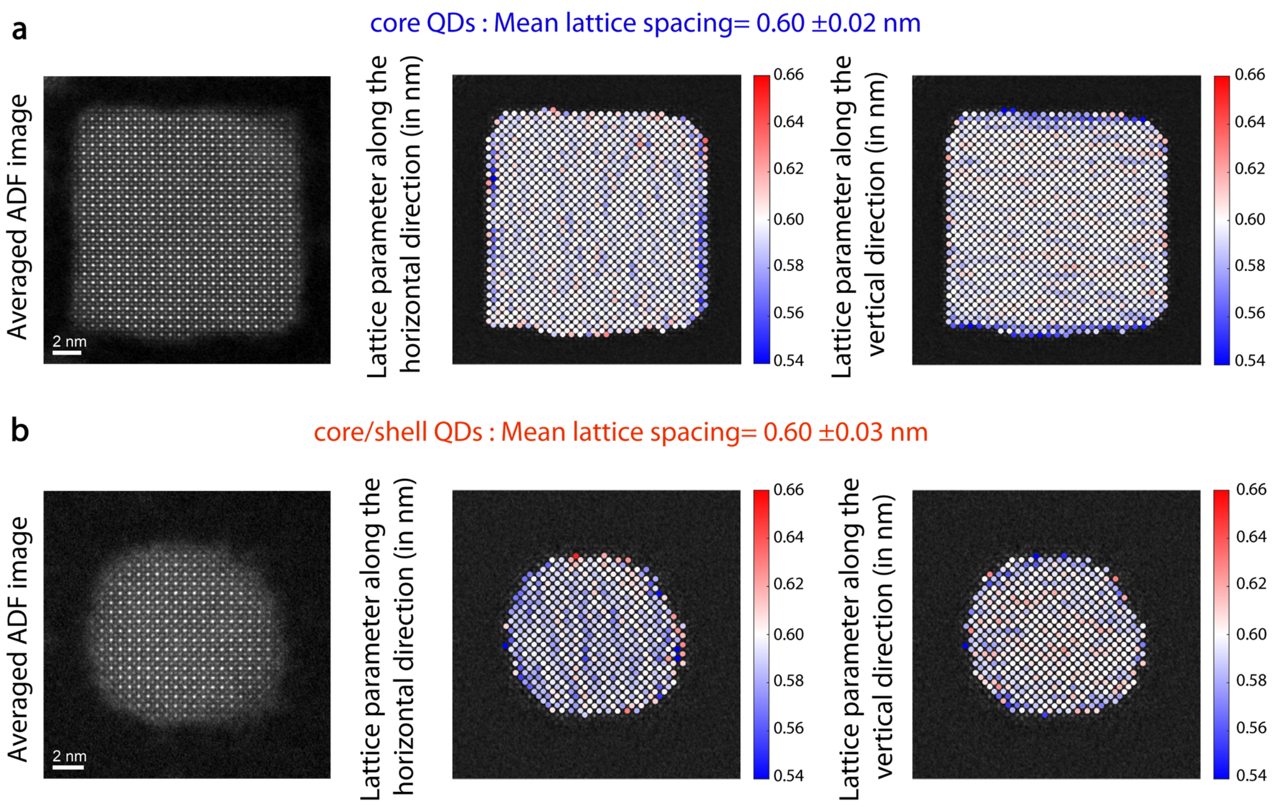

Supplementary Figure 14 | Structural analysis of core and core/shell QDs obtained via dilution. Lattice spacing estimation along the horizontal and vertical direction for core (**a**) and core/shell QDs (**b**), respectively. A mean lattice spacing of 0.6 nm has been found in both cases, suggesting that core/shell QDs do not have a built-in strain upon surface modifications.

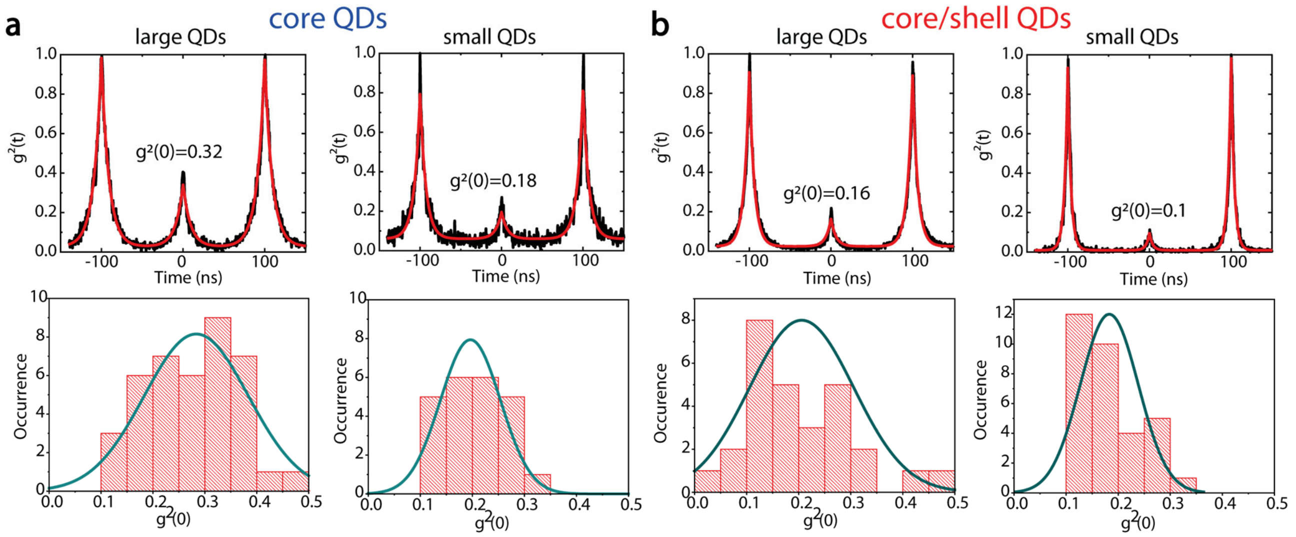

Supplementary Figure 15 | Photon statistics in core and core/shell QDs. Representative second-order correlation traces, and related statistical variation for several emitters, obtained for QDs with different edge length (large and small QDs, with 14 nm and 7 nm mean edge length) for core (**a**) and core/shell QDs (**b**), respectively.

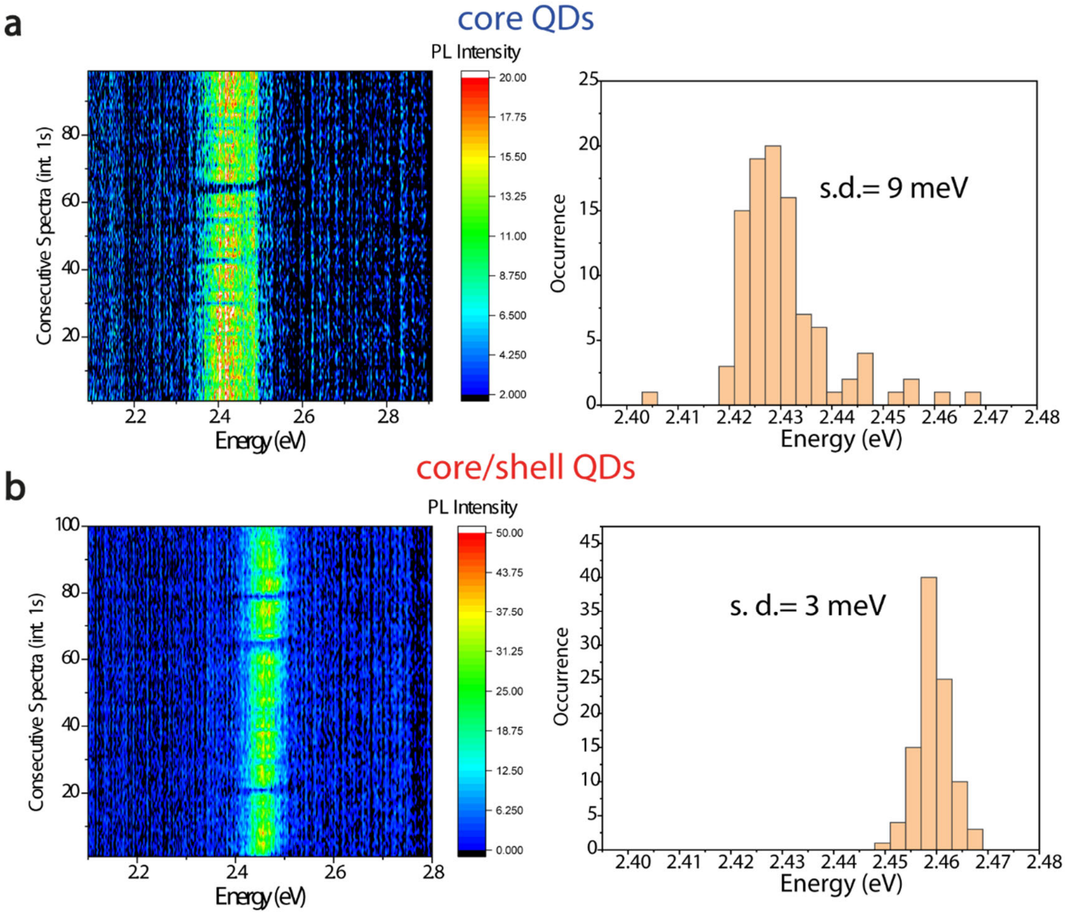

Supplementary Figure 16 | Spectral diffusion in core and core/shell QDs. The two-dimensional colored plots of several consecutive PL spectra obtained for core (**a**) and core/shell QDs (**b**). Spectral fluctuations are characterized by small standard variation (s.d.), much smaller than emission broadening.

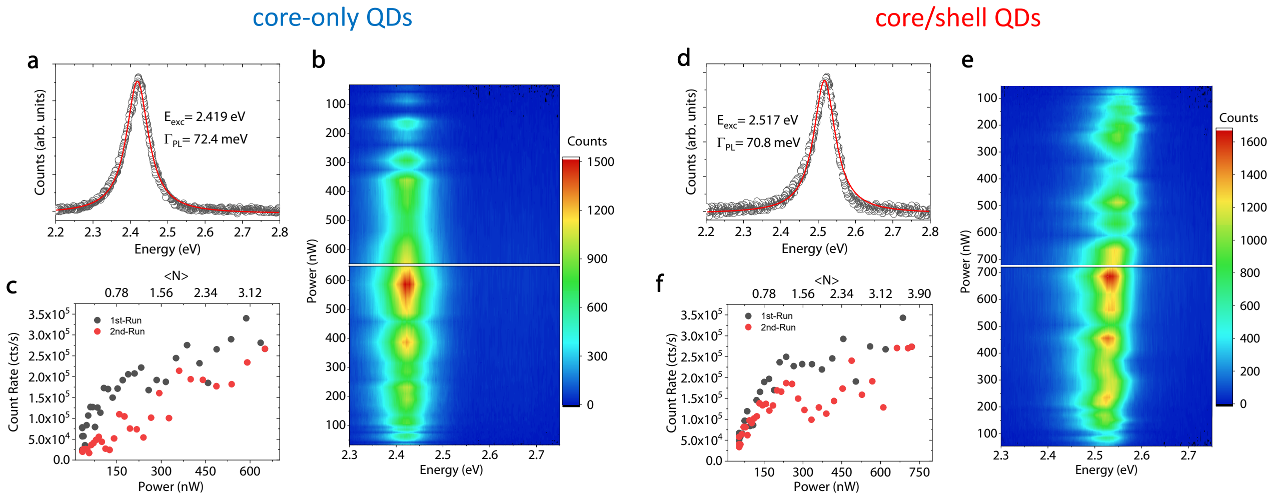

Supplementary Figure 17 | Excitation power-dependent PL experiments for core-only and core/shell QDs. a, PL spectrum of a core-only QD emitting at 2.419 eV with a PL FWHM of 72 meV. **b**, two-dimensional false-colour plots of PL spectra vs. excitation power, for two consecutive runs. **c**, spectrally-integrated PL count rate *vs.* excitation power, exhibiting a saturation level of about 2.5·10^5 counts/s. **d**, PL spectrum of a core/shell QD emitting at 2.517 eV with a PL FWHM of 71 meV. **e**, two-dimensional false-color plots of PL spectra *vs.* excitation power, for two consecutive runs. **f**, spectrally-integrated PL count rate vs. excitation power, exhibiting a saturation level of about 2-2.5·10^5 counts/s.

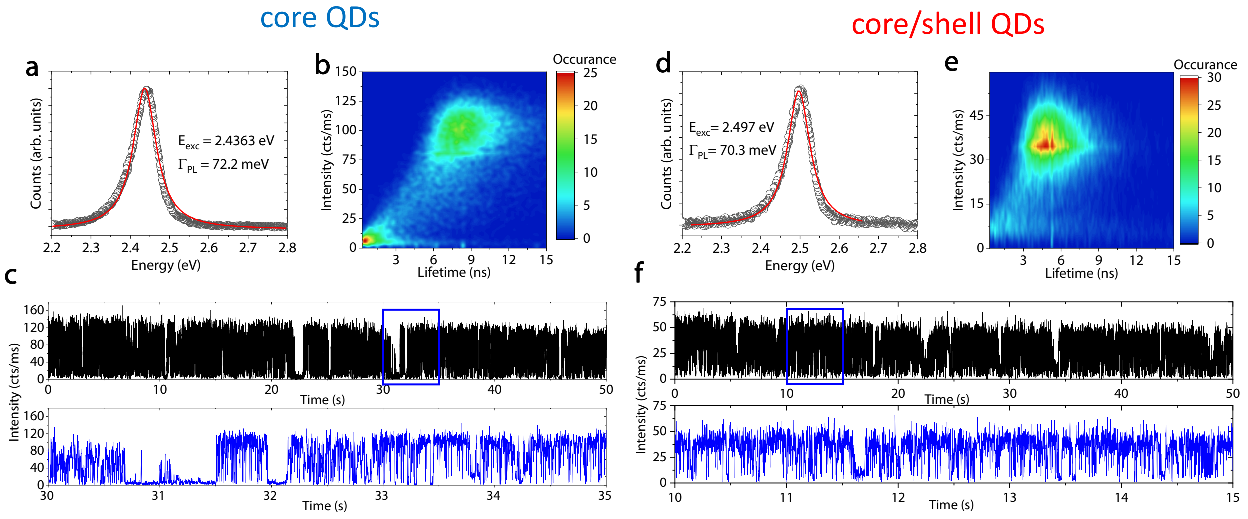

Supplementary Figure 18 | Blinking dynamic in core-only and core/shell QDs. **a**, PL spectrum of a core-only QD emitting at 2.4363 eV with a PL FWHM of 72.2 meV. **b**, The corresponding fluorescence-lifetime intensity distribution (FLID) colour plot. The two-dimensional histogram was constructed with a 0.1-ns lifetime binning and a 1 count intensity binning. The colour scale represents the frequency of occurrences of given intensity-lifetime pairs. **c**, *upper panel:* intensity histogram obtained with 1 ms binning; *lower panel*: blinking trace corresponding to the time window marked by a blue box in the upper panel. **d**, PL spectrum of a core/shell QD emitting at 2.497 eV with a PL FWHM of 70.3 meV. **e**, The corresponding core/shell QD FLID colour plot. The two-dimensional histogram was constructed with as in **b**. **f**, *upper panel:* intensity histogram obtained with 1 ms binning; *lower panel:* representative blinking trace corresponding to the time window marked by a blue box in the upper panel.

Supplementary References:

1. Yazdani, N. *et al.* Measuring the vibrational density of states of nanocrystal-based thin films with inelastic X-ray scattering. *J. Phys. Chem. Lett.* **9**, 1561-1567 (2018).
2. ten Brinck, S. & Infante, I. Surface termination, morphology, and bright photoluminescence of cesium lead halide perovskite nanocrystals. *ACS Energy Lett.* **1**, 1266-1272 (2016).
3. Brawand, N. P., Goldey, M. B., Vörös, M. & Galli, G. Defect states and charge transport in quantum dot solids. *Chem. Mater.* **29**, 1255-1262 (2017).
4. Rusishvili, M., Wippermann, S., Talapin, D. V. & Galli, G. Stoichiometry of the core determines the electronic structure of core–shell III–V/II–VI nanoparticles. *Chem. Mater.* **32**, 9798-9804 (2020).
5. Smith, B., Shakiba, M. & Akimov, A. V. Nonadiabatic dynamics in Si and CdSe nanoclusters: many-body vs single-particle treatment of excited states. *J. Chem. Theory and Comput.* **17**, 678-693 (2021).
6. VandeVondele, J. *et al.* Quickstep: fast and accurate density functional calculations using a mixed Gaussian and plane waves approach. *Comput. Phys. Commun.* **167**, 103-128 (2005).
7. Lippert, B. G., Parrinello, J. H. & Michele. A hybrid Gaussian and plane wave density functional scheme. *Mol. Phys.* **92**, 477-488 (1997).
8. Hartwigsen, C., Goedecker, S. & Hutter, J. Relativistic separable dual-space Gaussian pseudopotentials from H to Rn. *Phys. Rev. B* **58**, 3641-3662 (1998).

9 Bussi, G., Donadio, D. & Parrinello, M. Canonical sampling through velocity rescaling. *J. Chem. Phys.* **126**, 014101 (2007).

10 Besl, P. J. & McKay, N. D. A method for registration of 3-D shapes. *IEEE Trans. Pattern Anal. Mach. Intell.* **14**, 239-256 (1992).

11 Becker, M. A. *et al.* Bright triplet excitons in caesium lead halide perovskites. *Nature* **553**, 189 (2018).

12 Huang, K., Rhys, A. & Mott, N. F. Theory of light absorption and non-radiative transitions in F-centres. *Proc. R. Soc. Lond. A. Math. Phys. Sci.* **204**, 406-423 (1950).

13 Sercel, P. C. & Efros, A. L. Band-edge exciton in CdSe and other II–VI and III–V compound semiconductor nanocrystals − revisited. *Nano Lett.* **18**, 4061-4068 (2018).

14 Lin, S. H. *et al.* in *Adv. Chem. Phys.* 1-88 (2002).

15 Yazdani, N., Volk, S., Yarema, O., Yarema, M. & Wood, V. Size, ligand, and defect-dependent electron–phonon coupling in chalcogenide and perovskite nanocrystals and its impact on luminescence line widths. *ACS Photonics* **7**, 1088-1095 (2020).

16 Mukamel, S. Principles of nonlinear optical spectroscopy. *Oxford university press* **vol. 6.** (1995).

17 Yaffe, O. *et al.* Local polar fluctuations in lead halide perovskite crystals. *Phys. Rev. Lett.* **118**, 136001 (2017).

18 Wu, X. *et al.* Light-induced picosecond rotational disordering of the inorganic sublattice in hybrid perovskites. *Sci. Adv.* **3**, e1602388 (2017).

19 Bozyigit, D. *et al.* Soft surfaces of nanomaterials enable strong phonon interactions. *Nature* **531**, 618 (2016).

20 Campanini, M., Erni, R., Yang, C. H., Ramesh, R. & Rossell, M. D. Periodic giant polarization gradients in doped $BiFeO_3$ thin films. *Nano Lett.* **18**, 717-724 (2018).

21 Campanini, M. *et al.* Imaging and quantification of charged domain walls in $BiFeO_3$. *Nanoscale* **12**, 9186-9193 (2020).

22 Adams, R. Radial decomposition of disks and spheres. *CVGIP: Graphical Models and Image Processing* **55**, 325-332 (1993).

23 Du, H. A nonlinear filtering algorithm for denoising HR(S)TEM micrographs. *Ultramicroscopy* **151**, 62-67 (2015).

24 Watanabe, K. *et al.* Deconvolution processing of HAADF STEM images. *Ultramicroscopy* **92**, 191-199 (2002).

25 Yankovich, A. B. *et al.* Picometre-precision analysis of scanning transmission electron microscopy images of platinum nanocatalysts. *Nat. Commun.* **5**, 4155, doi:10.1038/ncomms5155 (2014).

26 Galindo, P. L. *et al.* The peak pairs algorithm for strain mapping from HRTEM images. *Ultramicroscopy* **107**, 1186-1193 (2007).

27 Erni, R., Heinrich, H. & Kostorz, G. Quantitative characterisation of chemical inhomogeneities in Al–Ag using high-resolution Z-contrast STEM. *Ultramicroscopy* **94**, 125-133 (2003).

28 Campanini, M. *et al.* Atomic-resolution differential phase contrast STEM on ferroelectric materials: A mean-field approach. *Phys. Rev. B* **101**, 184116 (2020).

29 Krieg, F. *et al.* Monodisperse long-chain sulfobetaine-capped $CsPbBr_3$ nanocrystals and their superfluorescent assemblies. *ACS Cent. Sci.* **7**, 135-144 (2021).

30 Williams, D. B. G. & Lawton, M. Drying of organic solvents: quantitative evaluation of the efficiency of several desiccants. *J. Org. Chem.* **75**, 8351-8354 (2010).
